# Echinoclerodane A: A New Bioactive Clerodane-Type Diterpenoid from a Gorgonian Coral *Echinomuricea* sp

**DOI:** 10.3390/molecules17089443

**Published:** 2012-08-07

**Authors:** Ching-Hsiao Cheng, Hsu-Ming Chung, Tsong-Long Hwang, Mei-Chin Lu, Zhi-Hong Wen, Yueh-Hsiung Kuo, Wei-Hsien Wang, Ping-Jyun Sung

**Affiliations:** 1 Department of Neurosurgery, Chang Gung Memorial Hospital-Kaohsiung Medical Center, Kaohsiung 833, Taiwan; Email: ma4200@adm.cgmh.org.tw; 2 Department of Marine Biotechnology and Resources and Asia-Pacific Ocean Research Center, National Sun Yat-sen University, Kaohsiung 833, Taiwan; Email: shiuanmin@yahoo.com.tw (H.-M.C.); wzh@mail.nsysu.edu.tw (Z.-H.W.); 3 National Museum of Marine Biology and Aquarium, Pingtung 944, Taiwan; Email: jinx6609@nmmba.gov.tw; 4 Graduate Institute of Natural Products, Chang Gung University, Taoyuan 333, Taiwan; Email: htl@mail.cgu.edu.tw; 5 Graduate Institute of Marine Biotechnology and Department of Life Science and Institute of Biotechnology, National Dong Hwa University, Pingtung 944, Taiwan; 6 Tsuzuki Institute for Traditional Medicine, China Medical University, Taichung 404, Taiwan; Email: kuoyh@mail.cmu.edu.tw

**Keywords:** *Echinomuricea*, clerodane diterpene, echinoclerodane, cytotoxicity, superoxide anion, elastase

## Abstract

A new clerodane-type diterpenoid, echinoclerodane A (**1**), was isolated from a Formosan gorgonian coral *Echinomuricea* sp. The structure of **1** was elucidated by spectroscopic methods. Echinoclerodane A (**1**) is the first clerodane-type compound obtained from the marine organisms belonging to the phylum Cnidaria. Echinoclerodane A (**1**) exhibited moderate cytotoxicity toward MOLT-4, HL-60, DLD-1 and LoVo tumor cells and inhibitory effects on the generation of superoxide anion and the release of elastase by human neutrophils.

## 1. Introduction

**Figure 1:**
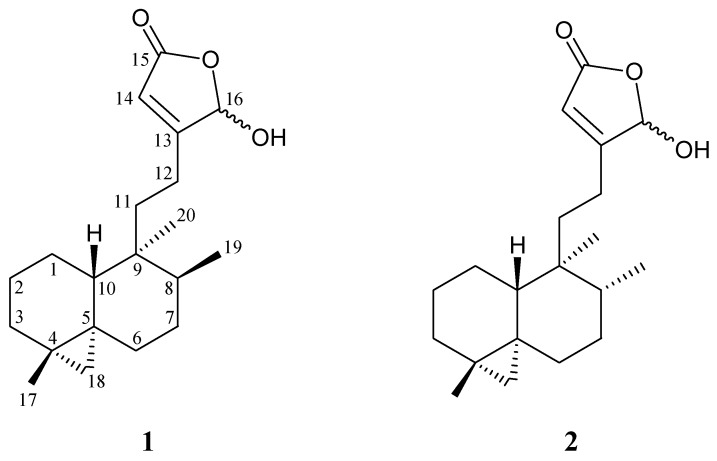
Structures of echinoclerodane A (**1**) and dytesinin A (**2**).

The search for bioactive natural products from marine organisms has been remarkably successful and gorgonian corals have proven to be rich sources of interesting natural terpenoid derivatives [1,2]. In previous studies, two bisabolane-type sesquiterpenoids, (7*S*,10*R*)-(+)-10,11-epoxycurcuphenol and (+)-curcuphenol; a labdane-type diterpenoid, echinolabdane A; and a steroid analogue, 6-*epi*-yonarasterol B, had been isolated from a Formosan gorgonian coral identified as *Echinomuricea* sp. (Plexauridae) [3,4]. In continuation of our search for new natural products from the marine invertebrates collected off the waters of Taiwan at the intersection of the Kuroshio current and the South China Sea surface current, we have further isolated a new clerodane-type diterpenoid, echinoclerodane A (**1**), from *Echinomuricea* sp. (Figure 1). In this paper, we describe the isolation, structure determination and biological activities of diterpenoid **1**.

## 2. Results and Discussion

Echinoclerodane A (**1**) was isolated as an oil and its molecular formula was determined to be C_20_H_30_O_3_ (*m/z* 341.2095 [M+Na]^+^) using HRESIMS. The IR spectrum of **1** showed bands at 3,318 and 1,741 cm^–1^, consistent with the presence of hydroxy and ester carbonyl groups. The ^13^C-NMR for **1** confirmed the presence of 20 carbon signals (Table 1), which were characterized by the DEPT spectrum as three methyls, eight sp^3^ methylenes, three sp^3^ methines, three sp^3^ quaternary carbons, one sp^2^ methine and two sp^2^ quaternary carbons. A suite of resonances at *δ*_C_ 171.8 (s, C-15), 171.2 (s, C-13), 116.9 (d, C-14) and 99.2 (s, C-16), could be assigned to an α,β-unsaturated-γ-hydroxy-γ-lactone moiety. Thus, from the reported data, the proposed skeleton of **1** was suggested to be a diterpenoid with four rings. 

**Table 1:** ^1^H- (400 MHz, CDCl_3_) and ^13^C- (100 MHz, CDCl_3_) NMR data, ^1^H–^1^H COSY and HMBC correlations for diterpenoid **1**.

Position	*δ*_H_ (*J* in Hz)	*δ*_C_, Mult.	^1^H–^1^H COSY	HMBC (H→C)
1a/b	0.75 dd (8.4, 2.8); 1.42 m	19.9, CH_2_	H_2_-2, H-10	C-9, -10
2a/b	1.19 m; 1.49 m	23.2, CH_2_	H_2_-1, H_2_-3	C-1, -3, -4
3	1.58 m	32.1, CH_2_	H_2_-2	C-4, -18
4		17.4, C		
5		26.3, C		
6a/b	1.02 m; 1.80 td (14.0, 2.8)	27.6, CH_2_	H_2_-7	C-5, -7, -8, -10, -18
7a/b	1.35 m; 1.92 tt (14.0, 4.0)	27.9, CH_2_	H_2_-6, H-8	C-6, -8, -19
8	1.68 m	35.6, CH	H_2_-7, H_3_-19	C-6, -7, -9, -19
9		39.1, C		
10	1.64 dd (12.4, 4.0)	40.9, CH	H_2_-1	C-8, -9, -10, -20
11	1.39 m; 1.59 m	35.5, CH_2_	H_2_-12	C-8, -9, -12
12	2.35 dd (8.8, 7.2)	21.5, CH_2_	H_2_-11, H-14	C-11, -13, -14, -16
13		171.2, C		
14	5.84 br s	116.9, CH	H_2_-12	C-12, -13,-15
15		171.8, C		
16	6.01 s	99.2, CH		C-13, -15
17	1.04 s	22.4, CH_3_		C-3, -4, -5
18a/b	0.13 d (4.4); 0.52 d (4.4)	24.5, CH_2_		C-3, -4, -5, -6, -10, -17
19	0.97 d (7.2)	14.2, CH_3_	H-8	C-7, -8, -9
20	1.00 s	19.8, CH_3_		C-8, -9

**Figure 2:**
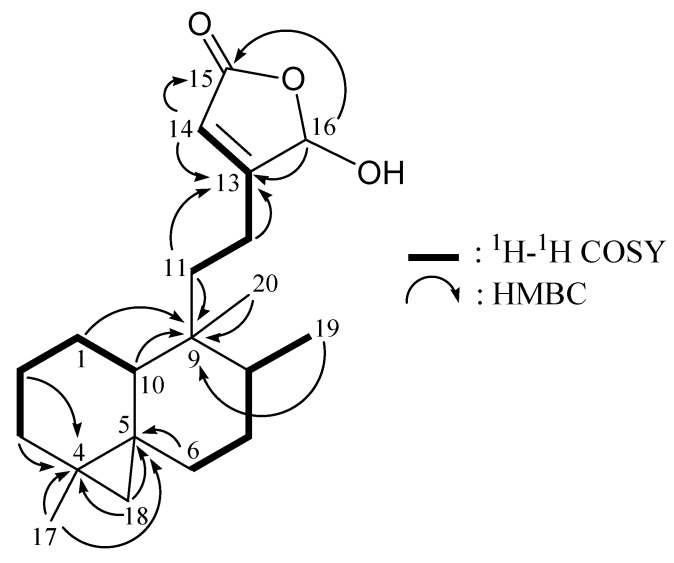
The ^1^H–^1^H COSY and selective key HMBC (protons→quaternary carbons) correlations for **1**.

From a ^1^H–^1^H COSY experiment (Table 1 and Figure 2), it was possible to establish the spin systems that map out the proton sequences from H-10/H_2_-1/H_2_-2/H_2_-3, H_2_-6/H_2_-7/H-8/H_3_-19, H_2_-11/H_2_-12 and H_2_-12/H-14 (by allylic coupling), which was accomplished with the assistance of an HMBC experiment (Table 1 and Figure 2).

The key HMBC correlations between the protons and quaternary carbons of **1**, including H_2_-2, H_2_-3, H_3_-17, H_2_-18/C-4; H_2_-6, H_3_-17, H_2_-18/C-5; H_2_-1, H-8, H-10, H_2_-11, H_3_-19, H_3_-20/C-9; H_2_-12, H-14, H-16/C-13; and H-14, H-16/C-15, permitted elucidation of the carbon skeleton. The tertiary methyls at C-4 and C-9 were confirmed by the HMBC correlations between H_3_-17/C-3, -4, -5 and H_3_-20/C-8, -9. The methine unit at *δ*_C_ 99.2 (d, C-16) was more shielded than expected for an oxygenated C-atom and correlated with a methine proton at *δ*_H_ 6.01 (H-16) in the HMQC spectrum, and this proton showed a ^2^*J*-correlation and a ^3^*J*-correlation with C-13 and C-15, respectively, in the HMBC spectrum and concluded to be a part of a hemiketal constellation.

**Figure 3:**
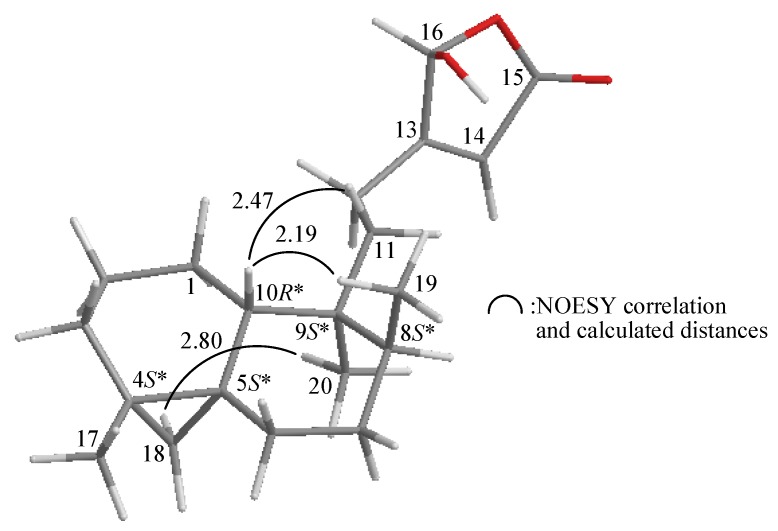
The computer-generated model of **1** using MM2 force field calculations and the calculated distances (Å) between selected protons with key NOESY correlations.

The relative configuration of **1** was elucidated mainly from a NOESY spectrum as being compatible with that of **1** offered by computer modeling [5], in which the close contacts of atoms in space calculated were consistent with the NOESY correlations (Figure 3). In the NOESY spectrum of **1**, the correlations of H-10 with H_2_-11 and H_3_-19, indicated that these protons (H-10, H_2_-11 and H_3_-19) were situated on the same face and these were assigned as β protons, since the C-20 methyl is an α-substituent at C-9. An *endo* H-C18 proton exhibited a correlation with Me-20, suggesting that the cyclopropane moiety between C-4/5 was α-oriented. Based on the above findings, the main structure of **1** was elucidated unambiguously, and the chiral carbons for **1** were assigned as 4S*, 5S*, 8S*, 9S*, 10R* although the relative configuration for 16-hydroxy group could not be determined at this stage by this method. By comparison of the spectral data, echinoclerodane A (**1**) was found to be the 8-epimer of a known marine-derived clerodane-type diterpenoid, dytesinin A (**2**) (Figure 1), isolated from an Okinawa tunicate *Cystodytes* sp. [6].

The cytotoxicity of diterpenoid **1** against the K562 (human erythromyeloblastoid leukemia), MOLT-4 (human acute lymphoblastic leukemia), HL-60 (human acute promyelocytic leukemia), DLD-1 (human colorectal adenocarcinoma), LoVo (human colorectal adenocarcinoma) and DU-145 (human prostate carcinoma) cells was studied, and the results were shown in Table 2. These data showed that echinoclerodane A exhibited moderate cytotoxicity against MOLT-4, HL-60, DLD-1 and LoVo cells. The *in vitro* anti-inflammatory effects of diterpenoid **1** were also tested. Echinoclerodane A (**1**) displayed a significant inhibition effect on the generation of superoxide anion (inhibition rate 68.6%) and this compound showed a moderately inhibition effect (inhibition rate 35.4%) on the release of elastase by human neutrophils at a concentration of 10 μg/mL, respectively [7].

**Table 2:** Cytotoxic activity of diterpenoid **1**.

Compounds	Cell lines IC_50_ (μM)					
	K562	MOLT-4	HL-60	DLD-1	LoVo	DU-145
**1**	37.05	13.18	14.89	23.44	21.69	53.93
Doxorubicin *^a^*	0.29	0.001	0.08	4.00	1.65	0.01

*^a^* Doxorubicin was used as positive control.

## 3. Experimental

### 3.1. General Experimental Procedures

Optical rotation values were measured with a Jasco-P1010 digital polarimeter. Infrared spectra were obtained on a Varian Diglab FTS 1000 FT-IR spectrophotometer. NMR spectra were recorded on a Varian Mercury Plus 400 FT-NMR at 400 MHz for ^1^H and 100 MHz for ^13^C in CDCl_3_ at 25 °C. Proton chemical shifts were referenced to the residual CHCl_3_ signal (*δ*_H_ 7.26 ppm). ^13^C-NMR spectra were referenced to the center peak of CDCl_3_ at *δ*_C_ 77.1 ppm. ESIMS and HRESIMS data were recorded on Bruker APEX II mass spectrometer. Column chromatography was performed on silica gel (230–400 mesh, Merck, Darmstadt, Germany). TLC was carried out on precoated Kieselgel 60 F_254_ (0.25 mm, Merck) and spots were visualized by spraying with 10% H_2_SO_4_ solution followed by heating. HPLC was performed using a system comprised of a Hitachi L-7100 pump, a Hitachi L-7455 photodiode array detector and a Rheodyne 7725 injection port. A normal phase column (Hibar 250 × 10 mm, Merck, silica gel 60, 5 μm) was used for HPLC.

### 3.2. Animal Material

Specimens of the gorgonian coral *Echinomuricea* sp. were collected by hand using scuba equipment off the coast of the southern Taiwan and stored in a freezer until extraction. This organism was identified by comparison with previous descriptions [8,9]. A voucher specimen (NMMBA-TW-GC-127) was deposited in the National Museum of Marine Biology and Aquarium, Taiwan.

### 3.3. Extraction and Isolation

The freeze-dried and minced material of *Echinomuricea* sp. (wet weight 1.68 kg, dry weight 428 g) was extracted with a 1:1 mixture of methanol (MeOH) and dichloromethane (CH_2_Cl_2_). The residue was partitioned with ethyl acetate (EtOAc) and H_2_O. The EtOAc phase was further partitioned between MeOH and *n*-hexane. The *n*-hexane phase was separated by silica gel and eluted using *n*-hexane/EtOAc/MeOH to yield 21 fractions A–U. Fraction N was separated on Sephadex LH-20 and eluted using a 1:1 mixture of MeOH/CH_2_Cl_2_ to yield 13 fractions. Fraction N3 was purified by NP-HPLC using a mixture of *n*-hexane and EtOAc (8:1, flow rate 5 mL/min) as the mobile phase to afford compound echinoclerodane A (**1**) (8.3 mg); oil; 
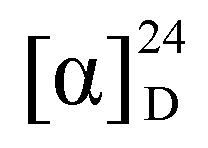
 −43 (*c* 0.07, CHCl_3_); IR (neat) ν_max_ 3,318, 1,741 cm^–1^; ^1^H- (CDCl_3_, 400 MHz) and ^13^C- (CDCl_3_, 100 MHz) NMR data, see Table 1; ESIMS *m/z* 341 [M+Na]^+^; HRESIMS: *m/z* 341.2095 (calcd. for C_20_H_30_O_3_Na, 341.2093).

### 3.4. Molecular Mechanics Calculations

The implementation of the MM2 force field [5] in the CHEM3D PRO software from CambridgeSoft Corporation (Cambridge, MA, USA; ver. 9.0, 2005) was used to calculate the molecular models.

### 3.5. Cytotoxicity Testing

The cytotoxicity of diterpenoid **1** was assayed with a modification of the 3-(4,5-dimethylthiazol-2-yl)-2,5-diphenyltetrazolium bromide (MTT) colorimetric method according to previously described procedures [10,11].

### 3.6. Superoxide Anion Generation and Elastase Release by Human Neutrophils

Human neutrophils were obtained by means of dextran sedimentation and Ficoll centrifugation. Superoxide generation and elastase release were carried out according to the procedures described previously [12,13]. Briefly, superoxide anion production was assayed by monitoring the superoxide dismutase-inhibitable reduction of ferricytochrome *c*. Elastase release experiments were performed using MeO-Suc-Ala-Ala-Pro-Valp-nitroanilide as the elastase substrate.

## 4. Conclusions

Clerodane-type diterpenoids are extensively present in terrestrial plants [14], and compounds of this type were also obtained from tunicates [6]. Octocorals have been proven to be rich sources of natural terpenoid derivatives and terpenoid analogues are often found in large amounts in marine invertebrates [15]. It is worth noting that the new clerodane metabolite **1** (echinoclerodane A) is the first clerodane-type derivative isolated from the marine organisms belonging to the phylum Cnidaria and this compound exhibited cytotoxicity and anti-inflammatory activity. The study material *Echinomuricea* sp. has begun to be transplanted to culturing tanks with a flow-through sea water system located in the National Museum of Marine Biology and Aquarium, Taiwan for the extraction of additional natural products in order to establish a stable supply of bioactive material.

*Sample Availability*: Not Available. 
